# Biofilm-inhibiting ZnO@Eggshell nanocomposites: green synthesis, characterization, and biomedical potential

**DOI:** 10.1007/s10534-025-00711-8

**Published:** 2025-07-02

**Authors:** Büşra Şensoy Gün, Rafig Gurbanov, Belgin Tunalı

**Affiliations:** 1 Central Research Laboratory Application and Research Center (BARUM), Bilecik Şeyh Edebali University, 11000 Bilecik, Türkiye; 2 Faculty of Science and Letters, Department of Nanoscience and Nanotechnology, Burdur Mehmet Akif Ersoy University, 2023 Burdur, Türkiye; 3 Faculty of Engineering, Department of Bioengineering, Bilecik Şeyh Edebali University, 11000 Bilecik, Türkiye

**Keywords:** Waste management, Eggshell, Antimicrobial, Green synthesis, Nanocomposite

## Abstract

This study explored the eco-friendly synthesis, characterization, optimization, and biomedical potential of zinc oxide-eggshell (ZnO@ES) nanocomposites using *Althaea officinalis* flower extract. HPLC analysis identified pink flower extract as the highest in quercetin (88.452 ppm), making it the optimal choice for synthesis. UV–Vis spectroscopy confirmed ZnO nanostructures (384 nm peak), while characterization analyses using different spectroscopic and microscopic techniques validated their successful incorporation within the eggshell matrix. The hemocompatibility of ZnO@ES nanocomposites was assessed through hemolysis tests, which demonstrated low hemolytic activity (<5%), ensuring blood compatibility. Antimicrobial assays against *Escherichia coli, Staphylococcus aureus, Pseudomonas aeruginosa,* and *Candida albicans* revealed significant inhibitory effects, particularly with ZnO@ES4. Agar well diffusion tests showed that while eggshell alone lacked antimicrobial activity, ZnO@ES2 formed inhibition zones against *P. aeruginosa* and *E. coli*, whereas ZnO@ES4 was effective against *P. aeruginosa*, *E. coli*, and *S. aureus*. Biofilm inhibition tests further demonstrated that ZnO@ES2 and ZnO@ES4 significantly reduced *E. coli* and *P. aeruginosa* biofilms, with ZnO@ES4 being more effective. MTT cytotoxicity assays using L929 fibroblast cells confirmed biocompatibility, with ZnO@ES2 enhancing cell proliferation. By repurposing eggshell waste, this study promotes a circular economy approach, transforming an abundant biowaste into value-added biomaterials. The green synthesis method eliminates the need for toxic chemicals, ensuring an environmentally friendly and sustainable clean production process. These findings support the development of antimicrobial and biocompatible nanocomposites with biomedical applications.

**Graphical Abstract:**

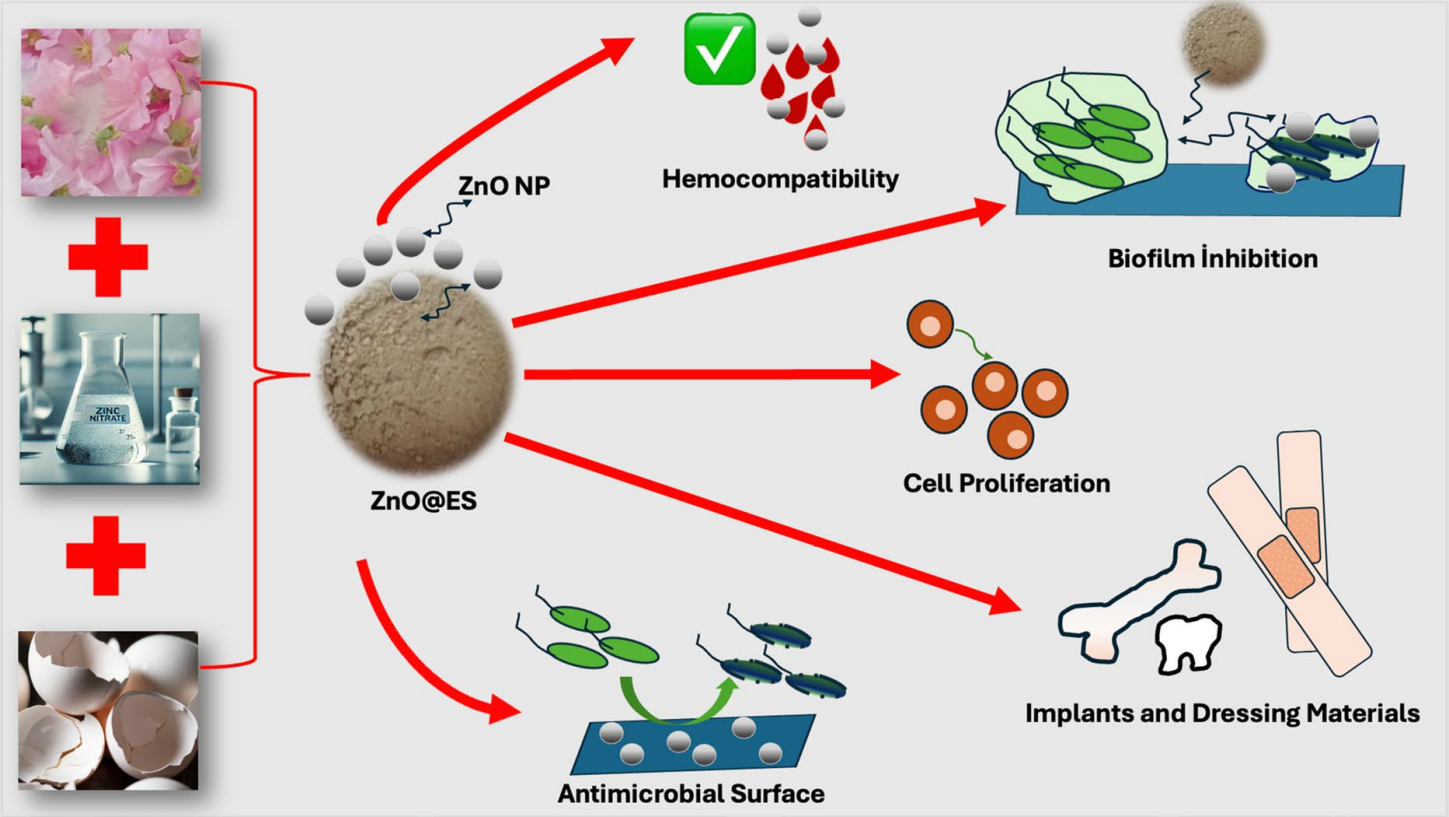

**Supplementary Information:**

The online version contains supplementary material available at 10.1007/s10534-025-00711-8.

## Introduction

In recent years, large-scale environmental problems have emerged due to the careless use of existing natural resources and the failure to consciously carry out waste management because of the rapidly increasing population. Hence, the use of wastes from natural resources and the production of bio-based materials are considered as a good solution. In addition, the use of low-cost, abundant, and easily obtained natural wastes also reduces production costs. Calcium carbonate has recently been preferred as a bio-based filling material. Chicken eggshell, which has a high calcium carbonate content, can be used as a potential and environmentally friendly alternative to inorganic (such as limestone) fillers in biomaterials (Owuamanam and Cree 2020; Tollu et al. 2024). The use of eggshells thrown into garbage as sustainable materials contributes to environmental waste management. In addition, ammonia, hydrogen sulfide, and bad odors originating from eggshells in garbage, and the emergence of pathogens that threaten human and animal health such as *Escherichia coli* and *Salmonella* can be reduced (Bee and Abdul 2020; Owuamanam and Cree 2020). The macro/micropores on the surface of the eggshell and the antimicrobial properties of the membrane part provide an advantage in its use as a good biomaterial (Martel et al. 2012). Current studies have revealed that nanocomposites synthesized using eggshell can be used as adsorbents, catalysts, photodegradation, tissue scaffolds, and in the production of antimicrobial nanomaterials (Li et al. 2016; Huang et al. 2020a; Gupta et al. 2021; Altuğ et al. 2022; Kınaytürk et al. 2021; Kalaycı et al. 2023; Tollu et al. 2024; Lopez et al. 2024).

New methods are being developed for the synthesis of metal nanoparticles, which possess a large surface area, unique optical properties, catalytic activity, and significant antimicrobial characteristics. These nanostructures are utilized in various fields, including imaging techniques, the medical industry, drug delivery, biosensors, and biomedical materials (Ijaz et al. 2020). Numerous methods exist for producing nanocomposites. However, recent studies have demonstrated that nanocomposites can now be synthesized in a single step using green synthesis methods (Huang et al. 2020b). Compared to other nanomaterial synthesis approaches, the green synthesis method has proven to be non-toxic, reliable, environmentally friendly, sustainable, highly reproducible, fast, suitable for large-scale production, and capable of producing more stable materials (El-Borady et al. 2021; Palithya et al. 2021; Yazdanian et al. 2022). On the other hand, plants with a high content of antioxidant compounds are known to be among the good candidates for nanoparticle synthesis. In syntheses performed using plants or plant parts (leaves, stems, and fruits), the reducing and stabilizing components found in plant extracts such as flavonoids, tannins, and phenols are selective and effective in regulating the size, shape, and distribution of nanoparticles (Maisa and Awwad 2021; Yazdanian et al. 2022). Quercetin, one of the preferred polyphenolic compounds in nanomaterial synthesis, easily forms complexes with various metals, preventing particle oxidation and imparting antibacterial properties to the particles (Mittal et al. 2014; Tasca and Antiochia 2020). The flowers of *Althaea officinalis*, a plant from the Malvaceae family that is widely available worldwide, possess therapeutic properties and have been used for years in traditional medicine for various ailments (Snafi 2013). Moreover, the flower parts of this plant are known to have high antioxidant content (Sadighara et al. 2012). Nanocomposites play a crucial role in various industries, particularly healthcare, due to their unique designable properties and customizable functions (Adeosun et al. 2012). Numerous studies have demonstrated that zinc oxide nanostructures and nanocomposite materials synthesized through green synthesis exhibit antimicrobial and wound-healing properties (George et al. 2019).

This research aimed to produce biocompatible nanocomposites with antimicrobial properties, utilizing eggshells as a bio-based material through a green synthesis approach. Among various plant extracts from the reddish-pink, white, and pink flower parts of *Althaea officinalis*, the extract with the highest quercetin concentration was identified using High-Performance Liquid Chromatography (HPLC). This selected extract was then combined with powdered eggshells and zinc nitrate solutions to create nanocomposites via the green synthesis technique. The characterization of the synthesized nanocomposites was conducted using a variety of analytical methods, including UV–Vis spectroscopy, Attenuated Total Reflectance Fourier Transform Infrared (ATR-FTIR) spectroscopy, X-ray Diffraction (XRD) spectroscopy, Scanning Electron Microscopy with Energy Dispersive X-ray Spectroscopy (SEM-EDX), Transmission Electron Microscopy (TEM) imaging, and Zeta potential analysis. The hemolytic and antimicrobial properties of the nanocomposites were thoroughly evaluated, and cytotoxicity assays were performed on the L929 fibroblast cells to assess the biocompatibility of the nanocomposites.

## Experimental

### Materials and chemicals

Eggshells were sourced from local markets. Mueller Hinton Broth, Mueller Hinton Agar, Tryptic Soy Agar, and Tryptic Soy Broth were purchased from Biolife. Zinc Nitrate Hexahydrate (TK.920085.01002), quercetin (337951-25G) extra pure, and PBS (PBS10X-05) were obtained commercially. Ethanol from Indosaw and acetone and methanol from Isolab were used in the study. Plant extracts and solutions were prepared using ultra-pure water obtained from the Zener 900 (UP: ≦18.3 MΩ·cm) device by Human Corporation.

### Methodology

#### Collection of *Althaea officinalis* plant

The reddish pink, white, and pink flower parts of *Althaea officinalis* plant were collected from the region of Burdur Province (Türkiye) at 37.726547 latitude and 30.25182 longitude in August. The collected flowers were washed in pure water and left to dry in a dark place at room temperature. The dried flowers were stored at + 4 °C to prepare the plant extract.

#### Preparation of plant extract from flower parts of *Althaea officinalis* plant

Two grams of dried and finely powdered flower parts were taken and transferred to separate 250 mL bottles containing 200 mL of deionized water and kept in a 60 °C water bath for 4 h. The resulting plant extract was filtered with Whatman No. 1 filter paper and centrifuged at 4500 rpm for 5 min. The obtained plant extracts were stored in a refrigerator at 4 °C for HPLC analysis (Ogunyemi et al. 2019, Adak et al. 2021, Abdallah et al. 2020).

#### HPLC analysis

High-performance liquid chromatography (HPLC) was used to calculate the quercetin amounts in plant extracts obtained from reddish-pink, white and pink flowers. Tarola et al. (2007) method was modified and used in HPLC analysis. SHIMADZU / UFLCXR Brand HPLC-DAD device, LC20 AT pump, and Tekno-Med-C18 RP (250*4.6 mm, 5 microns) column were used for analysis. The mobile phase was prepared as 5% acetic acid in acetonitrile. The sample with the highest quercetin amounts was selected and used in nanocomposite synthesis.

#### Preparation of eggshell powder

Eggshells were first washed under tap water to remove large dirt particles and then boiled in pure water at 120 °C for 3 h with continuous stirring to eliminate microbial contamination. The boiled eggshells were dried in a muffle furnace (~150 °C) for one day. The dried eggshells were ground into powder using an agate mortar and stored in a desiccator (Choudhary et al. 2021).

#### Nanocomposite synthesis

To prepare the nanocomposites, two grams of eggshells were added to 0.001 M and 0.01 M Zinc nitrate solutions (in ultrapure water) and stirred at 200 rpm for 12 h. Then, plant extract (zinc nitrate: plant extract: 9:1, 4:1 v/v) was added to the solutions and kept for 24 h. The resulting solution was centrifuged at 4500 rpm and the pellets were rinsed three times with distilled water and then kept in the oven at 60 °C overnight until dry (Huang et al. 2020a, b). The resulting nanocomposites were designated as presented in Table 1.

**Table 1:** Compositions of synthesized nanocomposites

Naming of nanocomposites	Zinc Nitrate (Molarity-M)	Eggshell (g)	Zinc nitrate: plant extract (v/v-vol/vol)
ZnO@ES1	0.001	2	4/1
ZnO@ES2	0.01	2	4/1
ZnO@ES3	0.001	2	9/1
ZnO@ES4	0.01	2	9/1

#### Characterization of nanocomposites

The presence of zinc oxide nanoparticles in the colloidal solution was confirmed by UV–Vis spectral analysis. The synthesis of nanoparticles can be observed qualitatively by the change in the color of the colloidal solution. Spectral analysis of nanocomposites consisting of different zinc nitrate and plant extract (v:v) mixtures was carried out at UV range of 300–400 nm (Abdallah et al. 2020). X-ray diffraction (XRD) analysis was performed using a Malvern panalytical empyrean device with Cu Ka radiation with a characteristic wavelength λ = 1.5406 Å and the data were obtained in the scanning range of 10° < 2θ < 90°. Attenuated total reflection Fourier Transform Infrared (ATR-FTIR) spectra were measured with a Spectrometer (Perkin Elmer Spectrum 100) in the 4000–400 cm^−1^ range with a resolution of 4 cm^−1^. The nanocomposites were coated by platinum sputtering on the QUORUM-Q ISORES instrument before morphology images and elemental mapping were obtained by Scanning electron microscopy (SEM) and energy dispersive X-ray spectroscopic analysis (EDX) using the ZEISS SUPRA 40VP GEMINI instrument. Zinc oxide nanostructures were also examined with the Hitachi HighTech HT7700 Transmission electron microscopy (TEM) instrument. Zeta potential of eggshell powder and nanocomposites were measured with MALVERN/NANO-ZS model device. To ensure homogeneous distribution, samples were dissolved with ultrapure water and sonicated. The measurements were carried out at 25 °C under ambient conditions. Three different measurements were taken for each sample, and the average value was calculated.

#### Evaluation of hemolytic activities

The Choudhary et al. (2021) method was optimized to determine the hemolytic activity of nanocomposite samples. Red Blood Cells (RBCs) were used for the experiment. A 1 mL of the blood sample was taken into the EDTA K3 tube and 25 mL of sterile saline (PBS) was added and centrifuged at 1000 rpm for 5 min. Then, the obtained RBCs were washed three times with 5 mL of PBS and dissolved in 50 mL of PBS. A 6 mg nanocomposite samples were added to 1.5 mL of RBC suspension and then incubated at 37 °C for 1 h. The mixture was centrifuged at 10.000 rpm and the supernatants were transferred to the UV cell and hemoglobin release was measured as absorbance (A) at 540 nm by UV spectrophotometry. Deionized water and phosphate-buffered saline (PBS) were used as positive and negative controls, respectively (Choudhary et al. 2021; Zhao et al. 2022). Percent hemolysis rates were calculated according to the following formula.

1 $$Hemolysis\% = \frac{(A)test\;sample - (A)negative\;control}{{(A)positive\;control - (A)negative\;control}} \times 100$$

#### Determination of minimum inhibitory concentration (MIC)

For MIC assays, *E. coli* ATCC 35218, *S. aureus* ATCC 25923, *P. aeruginosa* ATCC 27853, and *Candida albicans* ATCC 10239 were incubated overnight at 37 °C in Mueller-Hinton Broth (MHB) medium. After incubation, microbial suspensions were adjusted to a concentration of 5 × 10^5^ CFU/mL using a sterilized MHB medium. In sterilized 96-well microplates; 200 μL of solutions containing nanocomposite samples and eggshell powder prepared with sterile distilled water at a concentration of 4000 ppm were added to the first row. In the remaining wells, 100 μL of MHB was added. The samples were diluted using the serial dilution technique with a micropipette. Then, 10 μL of the suspension containing the respective microorganism was added to each well. The prepared microplates were incubated at 37 °C for 24 h, after which 10 μL of Resazurin solution (6.75 mg/mL) was added to each well. The experiments were performed in triplicate and incubated at 37°C for approximately 18–24 h. Visual observations of colorimetric changes, indicative of cellular viability, were conducted at hourly intervals. The transition from purple to pink was monitored colorimetrically, and absorbance values were measured at 600 nm using a microplate reader (Multiskan FC, Thermo Scientific). Control groups were set as dye + culture, dye, and dye + MHB (Ogunyemi et al. 2019; Selvam et al. 2020).

#### Evaluation of Antibacterial Activities

The antibacterial activities of nanocomposites against pathogenic bacteria were examined by Kirby-Bauer, well-diffusion method*. E. coli* ATCC 35218, *S. aureus* ATCC 25923, and *P. aeruginosa* ATCC 27853 test strains were incubated in TSB (Tryptic Soy Broth) for 24 hours at 37°C. After incubation, the bacterial culture was diluted to a concentration of 1.5 × 10^6^ CFU.mL^−1^ in TSB. Bacterial suspensions were spread on the agar plate surface under aseptic conditions using sterile cotton swabs. A 40 μL of the concentration of nanocomposites (mg/mL) determined according to the MIC test result was added to the wells with a diameter of 6 mm in agar plates and incubated at 37 °C for 24 h. Antibacterial activity was evaluated by determining the diameter of the net inhibition zone around the well expressed in millimeters (mm) (Suwan et al 2018).

#### Evaluation of antifungal activities

The antifungal activities of nanocomposites against *C. albicans* ATCC 10239 were examined by Kirby-Bauer, well-diffusion method. Pure culture was incubated in MHB (Mueller Hinton Broth) medium for 24 h at 37 °C. After incubation, bacterial culture was diluted to a concentration of 1.5 × 10^6^ CFU.mL^−1^ in MHB, and fungal culture was spread on MH agar (Following CLSI M44-A2; Mueller Hinton Agar + 2% glucose and 0.5 microgram/mL methylene blue) plate surface under aseptic conditions using sterile cotton swabs. A 40 μL of the concentration of nanocomposites determined according to the MIC test result (mg/mL) was added to the wells with a diameter of 6 mm in agar plates and incubated at 37 °C for 48 h. Antifungal activity was evaluated by determining the diameter of the net inhibition zone around the well expressed in millimeters (mm) (Suwan et al 2018).

#### Determination of anti-biofilm activities

To examine the biofilm activity of nanocomposites against *E. coli* ATCC 35218 *and P. aeruginosa* ATCC 27853 strains having the ability to form biofilms, the bacteria were incubated in TSB for 24 h at 37 °C. After incubation, bacterial suspension was adjusted to have a cell density of 1 × 10^6^ at OD_600_ and 100 μL from adjusted suspension were seeded in 96 well plates. A 100 μL of different concentrations of nanocomposites (4, 2, 1, and 0.5 mg/mL) were added to the bacteria in the wells by making serial dilutions and incubated overnight at 37 °C. After incubation, the culture medium in the 96 well plate was removed and the wells were washed with water 3 times. After adding methanol to the washed wells, they were left for fixation for 15 min. After removing the methanol, 130 μL of 0.1% prepared crystal violet dye solution was added to the dried wells and left for 15 min. The wells were washed with water 3 times to remove crystal violet. Finally, 30% acetic acid solution was added to the wells, and absorbance was measured at OD_550_ using a microplate reader (Multiskan FC, Thermo Scientific) (Al-Ogaidi et al. 2024).

#### Evaluation of cytotoxicity using MTT assay

To evaluate the biocompatibility of nanocomposites, cytotoxicity tests were performed on L929 fibroblast cells using 3-(4,5-dimethylthiazol-2-yl)-2,5-diphenyltetrazolium bromide (MTT) reduction assay. Cells were cultured in 10% fetal bovine serum and 1% penicillin-streptomycin medium at 37 °C in humidified air with 5% CO_2_. A volume of 1 mL of cell suspension was seeded into a 96-well plate and incubated overnight, then eggshell and nanocomposite samples (0, 0.25, 0.5, 1, 2, 4, and 8 mg/mL) were seeded into the 96-well plate at the determined concentration (approximately 3 × 10^6^ cells/mL per well). After 24 h incubation, 10 μL of MTT reagent was added to each well. The cells were incubated for another 3 h and cell viability was assessed compared to untreated control cells by measuring  the absorbance at 570 nm using a microplate reader (Multiskan FC, Thermo Scientific) (Long et al. 2017).

#### Statistical analyses

Analyses were performed using the statistical analysis program GraphPad Prism 10.01 (GraphPad Software, San Diego, California). Dunnett's multiple comparison test in a two-way ANOVA test was used to determine the statistical significance of numerical variables between groups. Results are shown as mean value ± standard error of the mean (SEM). The significance level was always determined as * p ≤ 0.05, ** p ≤ 0.01, *** p ≤ 0.001, **** p ≤ 0.0001 at 95% confidence interval. Non-significant p-values are presented without any asterisks.

## Results and discussion

### Chemical and morphological characterization of synthesized nanocomposites

Nanocomposite synthesis often involves the selection of plant extracts based on their bioactive compound content, with quercetin being a key determinant due to its significant role in enhancing material properties.

The plant extract to be used in the nanocomposite synthesis was selected by determining different quercetin amounts depend on the HPLC analysis results (Fig. S1). According to the HPLC analysis results given in Table 2, the amount of quercetin was found to be 62.799, 82.225, and 88.452 ppm in the plant extracts prepared from reddish-pink, white, and pink flower parts, respectively.

**Table 2.** Quantities of quercetin in plant extracts obtained from *Althaea officinalis*

Plant extracts	Ret. time	Peak area	Peak height	Quercetin concentration (ppm)
Reddish pink	4.052	2467723	175976	62.799
White	4.039	297679	230412	82.225
Pink	4.034	2835548	247863	88.452

There are studies in the literature that quercetin flavonoid plays an important role in the reduction of metal ions, stabilization of the formed nanoparticles and increasing their antimicrobial activity (Mittal et al. 2014; Sarıbuğra 2014; Shah et al. 2017; Tasca and Antiochia 2020; Lafta et al. 2025). According to density functional theory (DFT) analyses, the dissociation energy of –OH groups, especially in the catechol structure of flavonoids, is low (Leopoldini et al. 2004; Trouillas et al. 2006). This allows these regions to become more reactive in redox reactions and to play a selective role in the dissociation of metal ions. Quercetin molecule has the ability to reduce metal ions such as Zn^2^⁺, Au+ and Ag⁺, thanks to the phenolic –OH groups in its structure and the reactive hydrogen atoms released during enol–keto tautomerization (Bollella et al. 2016; Tasca and Antiochia 2020; Lafta et al. 2025). For this reason, we preferred to use the plant extract with the highest quercetin content in our study in the synthesis of nanocomposites. Our HPLC analysis reveals that the quercetin concentration in the pink flower extract of *Althaea officinalis* exceeds that found in other flower extracts, leading to the selection of the pink flower extract for the synthesis of nanocomposites. It is thought that this natural reducing agent will reduce the Zn^2^⁺ ions in the Zn(NO₃)₂ solution, while at the same time ensuring the adhesion of the formed ZnO nanoparticles to the eggshell, thus increasing the homogeneous formation and stability. In addition, flavonoid structures such as quercetin that remain adsorbed on the surface show an additional biological function by enhancing the antimicrobial activity of ZnO@ES nanocomposites. The high reduction potential of quercetin with this information is aimed to increase the performance of this process by thermodynamically enabling the conversion of Zn^2^⁺ ions into ZnO nanoparticles.

In this study, a single-step, environmentally friendly, and sustainable green synthesis was carried out to produce nanocomposite containing zinc oxide nanoparticles using pink flower extract of *Althaea officinalis* plant and eggshell powder. For this purpose, the pink flower extract of *Althaea officinalis* plant was used to produce NPs in the synthesis. During the process of green synthesis, various components such as flavonoids, enzymes, antioxidants, and phenolic compounds, including zinc nitrate and nitrate-reducing compounds, play a crucial role in the formation of zinc oxide. In this phytochemical reaction, zinc nitrate cations undergo reduction, leading to the formation of zinc oxide nanoparticles. These nanoparticles are stabilized by certain phenolic compounds, notably quercetin (Patil and Taranath 2016; Verma et al. 2020; Aljabali et al. 2022). Initially, the flower extract exhibits a pink hue, transitions to a yellowish color within approximately 30 min, ultimately evolving into a greenish-light brown shade by the end of the incubation period. This observable color change serves as an indicator of the successful synthesis of zinc oxide nanoparticles (Abdo et al. 2021). To further validate the formation of zinc oxide nanoparticles, UV–Vis spectroscopic analysis was conducted. The results, illustrated in Fig. S2, reveal a characteristic absorption peak at 384 nm, which is indicative of the presence of zinc oxide nanoparticles (Abdo et al. 2021; Aljabali et al. 2022). Consequently, both the observed color change and the UV–Vis analysis confirm the successful synthesis of zinc oxide nanoparticles during the green synthesis process.

The infrared (IR) spectra of nanocomposites synthesized from the pink flower parts of *Althaea officinalis* and eggshell powder are given in Fig. S3. It should be noted that the eggshell powder used in this study was obtained without separating the shell and membrane of the eggshell. The aim here is to ensure that the collagen-like structures and proteins in the membrane part can be incorporated into the structure without being disrupted during the synthesis of the nanocomposite. In this way, the produced nanoparticles were not only attached/interacted with the eggshell but also with the fibrous structures in the membrane part. As can be seen from the bands in the IR spectrum of the eggshell powder, the protein structures in the membrane part were preserved (Fig. S3a). Moreover, the absorption bands related to antisymmetric stretching, out-of-plane bending, and in-plane vibrations related to calcite in the structure were observed at the positions of 1393, 1040, 872, and 712 cm^−1^, respectively (Mittal et al. 2014; Şensoy Gün et al. 2024). On the other hand, the bands at 3402 and 1646 cm^−1^ are characteristic for amide bonds in the membrane structure of the eggshell (Mansilla and Mejia 2017; Chen et al. 2022). The absorption band at 2515 cm^-1^ shows the thiol bonds of cysteine in the intact membrane (Bhagavatheswaran et al. 2019). The bands at 2957 and 2828 cm^−1^ come from symmetric and antisymmetric carbon–hydrogen bonds originating from both the minerals in the shell part and the proteins and lipids in the membrane structure (Mittal et al. 2014; Mansilla and Mejia 2017; Parvin et al. 2019).

When the spectra of zinc oxide nanoparticles immobilized in eggshell powder were examined, no change was observed except for the intensities in the absorption bands associated with calcite and membrane structures. This indicates that the nanoparticles were successfully immobilized in eggshell powder and did not interact with calcite and membrane structures (Nasrollahzadeh et al. 2016; Yorseng et al. 2020). The bands around 600 and 400 cm^−1^ in the IR spectrum are attributed to the metal-oxygen (MeO) vibration mode (He et al. 2019; Ruhaimi and Aziz 2021; Ashwini et al. 2021), while the absorption bands at 480 and 499 cm^−1^ are due to the metal-oxygen stretching mode of zinc oxide (Selvam et al. 2020; Mutukwa et al. 2022). The changes observed in each of the intensity and wavenumber of the zinc oxide bands in the nanocomposite samples occur as a result of different structural interactions between zinc oxide and eggshells. In the ZnO@ES3 nanocomposite, zinc oxide nanoparticles penetrate less into the eggshell powder compared to other nanocomposites. This is observed as a lower zinc oxide band intensity in the IR spectrum, because the synthesis of ZnO@ES3 was carried out using 0.001 M zinc nitrate solution and minimum amounts of plant extract. In many studies, parameters such as temperature, pH, metal salt, and reaction time were optimized for green synthesis, and it was determined that optimization process affects many characteristics of the nanoparticles such as shape, amount, and antimicrobial properties (Hoseinpour et al. 2017; Anvarinezhad et al. 2020). Therefore, the quantity of plant extract and the molarity of zinc nitrate utilized significantly influence the synthesis of zinc oxide nanoparticles through green methods. Consequently, variations in the intensities of the zinc oxide bands were noted in the IR spectrum, as illustrated in Fig. S3b.

**Fig. 1:**
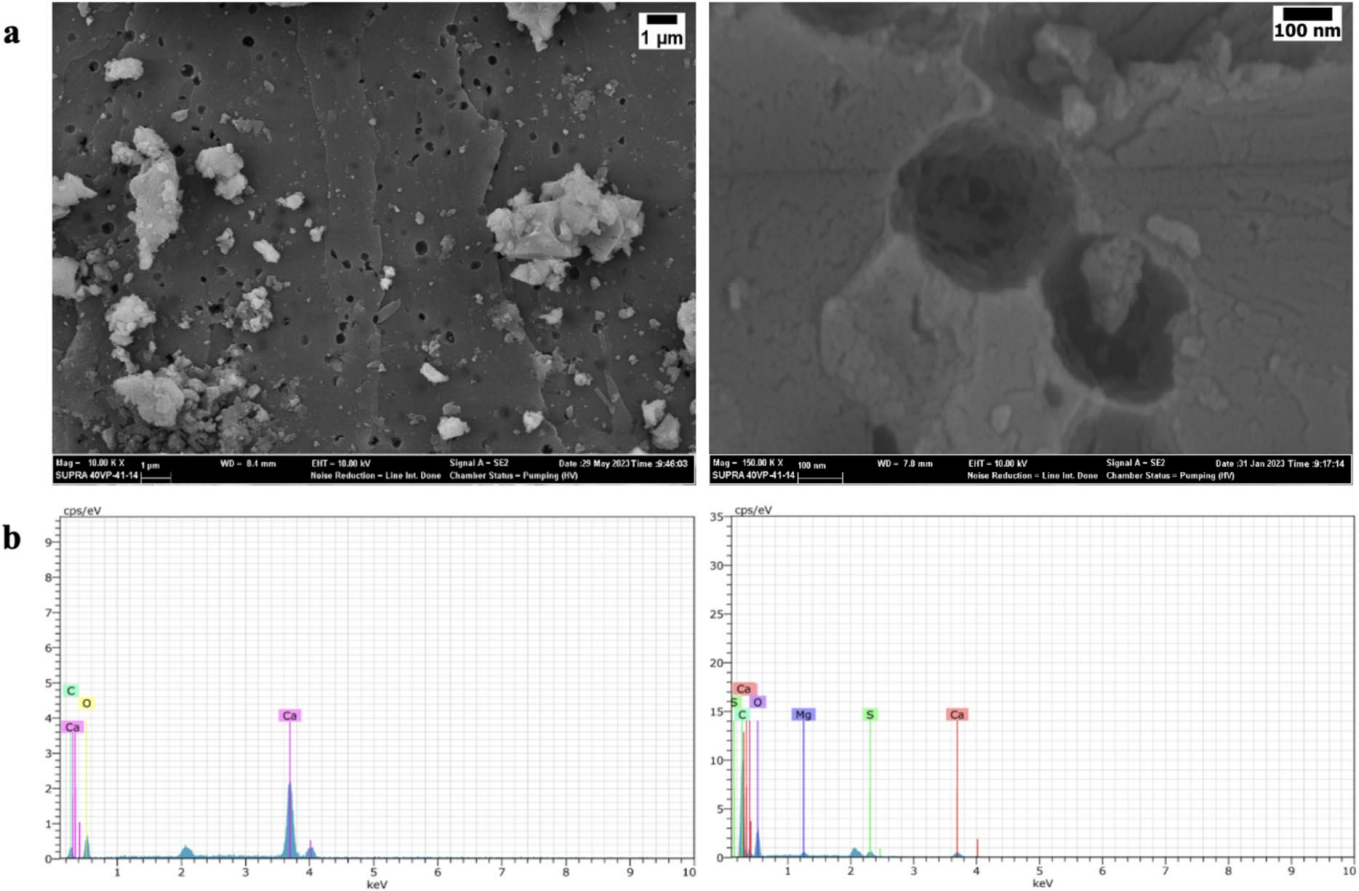
**a** Scanning Electron Microscopy (SEM) images of empty eggshell powder (ES) highlighting both macro and micropore features. **b** Energy-dispersive X-ray (EDX) spectra of empty eggshell powder (ES)

Calcite structures and macro-micropore structures inherent in the empty eggshell surface are seen in the SEM image given in Fig. 1. The natural pores of the eggshell are approximately 200 nm in size (Fig. 1a). Moreover, Fig. 1b shows EDX spectra taken from at least two different points on the surface of the eggshell powder. The analysis of the EDX spectrum of the eggshell reveals that its elemental composition is predominantly comprised of calcium (Ca), carbon (C), and oxygen (O). Additionally, due to the use of eggshell powder that includes both the shell and membrane, the EDX spectrum also indicates the presence of sulfur (S) and magnesium (Mg) elements originating from the membrane part (Kumar et al. 2018; Honarmand et al. 2020).

**Fig. 2:**
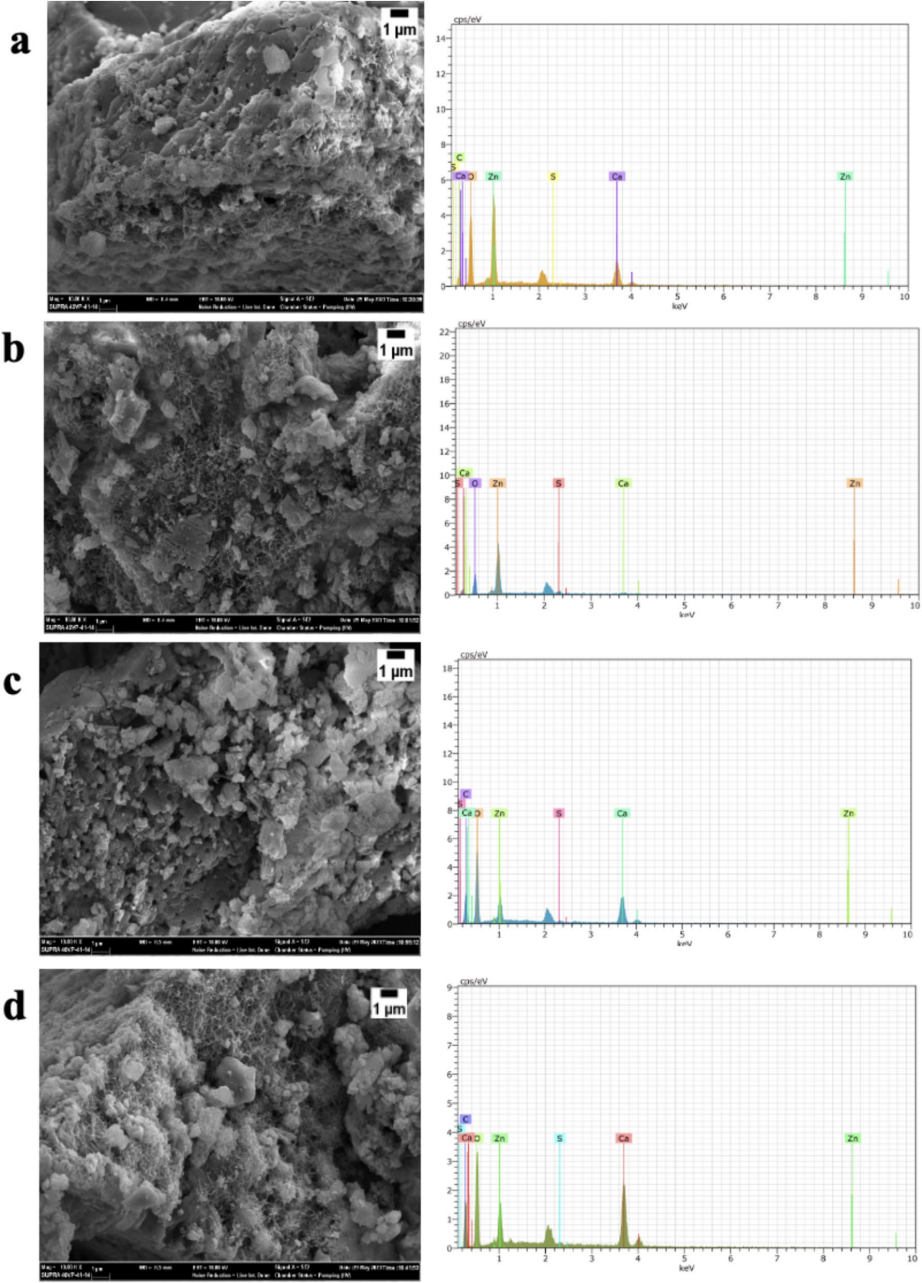
Scanning Electron Microscopy (SEM) images and EDX spectra of synthesized nanocomposites **a** ZnO@ES1, **b** ZnO@ES2, **c** ZnO@ES3, and **d** ZnO@ES4

In this study, it was planned to incorporate zinc oxide nanoparticles onto the surface of eggshells by green synthesis method. The morphologies of eggshell powder and nanocomposites, as well as the size and shape of zinc oxide structures, were determined by SEM analysis. As seen in Fig. 2, the porous structures of the eggshell were coated with zinc oxide nanoparticles. Zinc oxide nanorods, nanoparticle structures, and nanoflakes were formed on certain areas of the eggshell surface (Fig. 2a–d). Different zinc oxide morphologies are observed on the surfaces of the nanocomposites formed after green synthesis. While nanoparticle, nanorod, and nanoflake structures of zinc oxide are observed on the surfaces of the ZnO@ES1, ZnO@ES2, and ZnO@ES4 nanocomposites, single-type zinc oxide nanoparticles are observed in ZnO@ES3 nanocomposite (Fig. S4a). The observed characteristics of the ZnO@ES3 nanocomposite can be attributed to the varying volumes and molarities of plant extract and zinc nitrate utilized during the green synthesis process. The diameter of the zinc oxide nanoparticles deposited on the eggshell surface was measured to be approximately in between 30 and 50 nm, while the diameter of the nanorods was measured to be approximately in between 24 and 50 nm (Fig. S4b). The lengths and diameters of the nanoparticles and nanorods vary, but it was confirmed by both SEM and XRD analyses that zinc oxide was produced in hexagonal morphology. To ensure that zinc oxide nanoparticles are highly effective in nanomedicine applications and can interact with cells, their sizes should ideally be below 200 nm (Rizvi and Saleh 2018). This study suggests that the particle sizes of the nanostructures in the synthesized nanocomposites, as observed through SEM and TEM analyses, are appropriate for nanomedicine applications and possess the potential for cellular interaction.

During the characterization of nanocomposites obtained via green synthesis, elemental analysis was performed to detect the presence of zinc oxide. EDX analysis was conducted on point-specific and regionally selected areas during SEM imaging. When comparing the EDX spectra of the nanocomposites presented in Fig. 2 with the EDX spectra of the eggshell shown in Fig. 1b, the presence of zinc oxide formed on the eggshell surface during green synthesis was confirmed. These EDX data strongly support the successful realization of the green synthesis process.

**Fig. 3:**
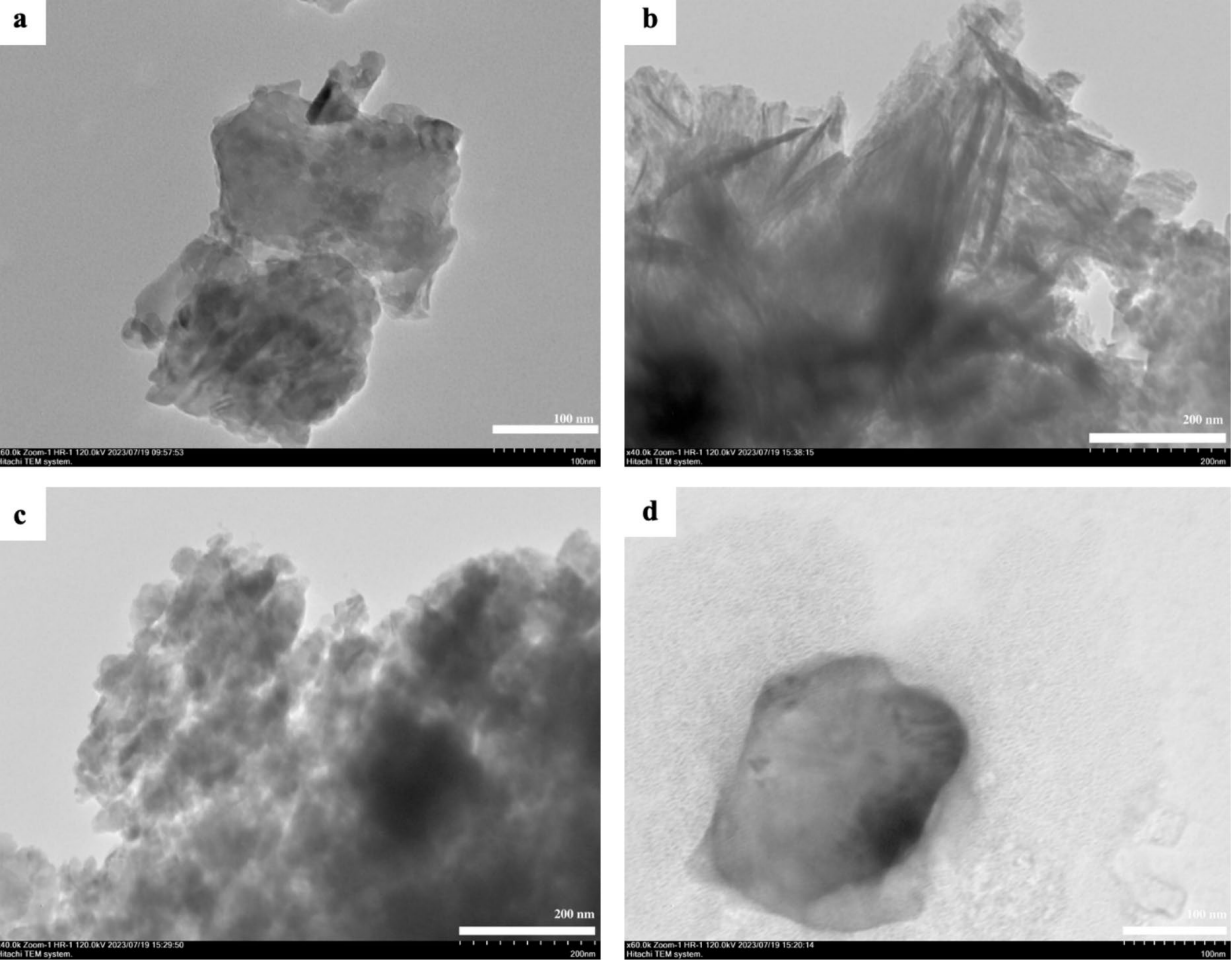
Transmission electron microscopy (TEM) images of synthesized nanocomposites **a** ZnO@ES1, **b** ZnO@ES2, **c** ZnO@ES3, and **d** ZnO@ES4

The TEM analysis results of the nanocomposites are given in Fig. 3 for better characterization of microstructures in the ZnO@ES nanocomposites. The results of the TEM analysis revealed distinct zinc oxide structures formed on eggshell powder (Fig. 3a–d). Notably, Fig. 3b illustrates the presence of nanorods within the nanocomposite structure. Additionally, the TEM images indicate the presence of smaller nanorods with nanometer diameters, as well as nanoparticle structures, which align with the findings from SEM imaging. However, the porous structure of the eggshell is not discernible in the TEM images, potentially due to the sample's opacity (Qian et al. 2021). The observed formation of nanoparticles, nanoflakes, and predominantly needle-like zinc oxide nanorods in both TEM and SEM images underscores the significant influence of the unique biomolecular composition derived from the pink flower parts of the *Althaea officinialis* plant utilized in this study. This combination played a crucial role in achieving the distinctive shapes of the nanoparticles. In contrast to conventional methods that typically employ strong bases, surface-active agents, or catalysts for the synthesis of zinc oxide nanorods (Awad et al. 2024), our research adopted a more environmentally friendly approach. The production of zinc oxide nanorods was accomplished through a sustainable and non-toxic green synthesis method, eliminating the need for harmful chemical substances.

**Fig. 4:**
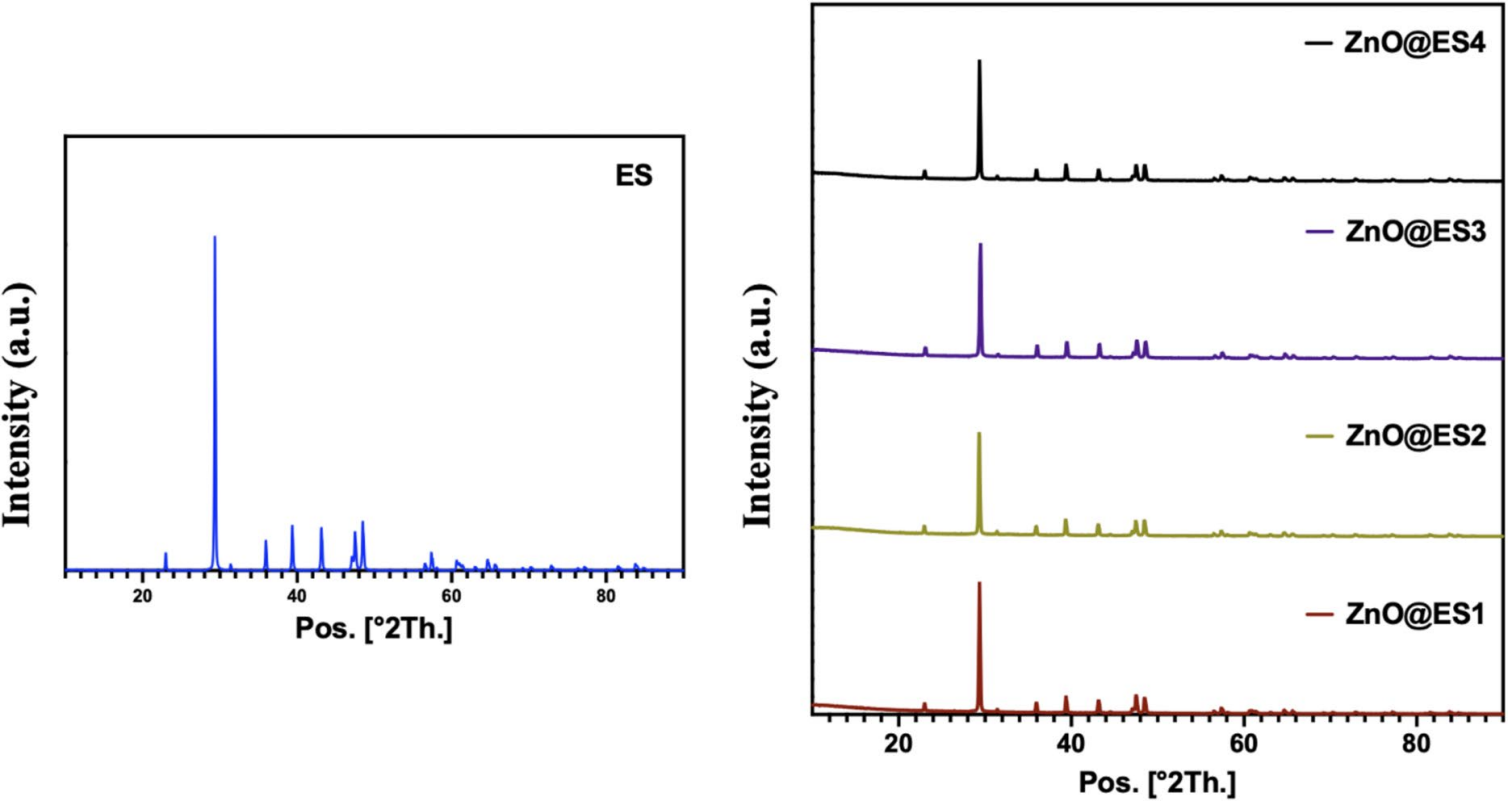
XRD diffraction patterns of eggshell powder (ES) and ZnO@ES nanocomposites

X-ray diffraction (XRD) analysis was conducted to determine the crystalline structure of the synthesized nanocomposites and eggshell powder. Based on the library scans of the XRD patterns shown in Fig. 4, the characteristic peaks of CaCO_3_ at 2θ = 29.37, 39.38, 43.13, 47.49, and 48.49 (CaCO₃, card no. 96-901-6707) were identified for eggshell powder. Meanwhile, the XRD diffraction patterns of ZnO@ES1, ZnO@ES2, and ZnO@ES4 nanocomposites revealed the characteristic peaks of ZnO at 2θ = 31.27, 35.86, 47.32, 62.81, 65.24, 75.99, and 81.52, while the ZnO@ES3 nanocomposite exhibited ZnO peaks at 2θ = 31.27, 35.86, 47.32, 65.24, and 75.99 (ZnO, card no. 98-018-1039) (Kalpana et al. 2018; Qian et al. 2021). The FTIR and SEM analyses indicated that the ZnO@ES3 nanocomposite exhibited a lower concentration of zinc oxide nanoparticles integrated into the eggshell powder matrix when compared to the other synthesized nanocomposites. Moreover, the examination of the XRD diffraction patterns demonstrated that the characteristic peaks of zinc oxide were less prominent in ZnO@ES3 relative to the other nanocomposites. This finding reinforces the consistency of results across the various characterization techniques employed. Furthermore, supplementary diffraction peaks corresponding to the pure phases of ZnO and CaCO_3_ were observed in the XRD patterns of all nanocomposites. Certain differences were noted in the XRD patterns of the nanocomposites compared to that of eggshell powder, indicating structural modifications. These findings demonstrate that zinc oxide nanoparticles formed during the green synthesis process were successfully conjugated onto the eggshell surface.

**Fig. 5:**
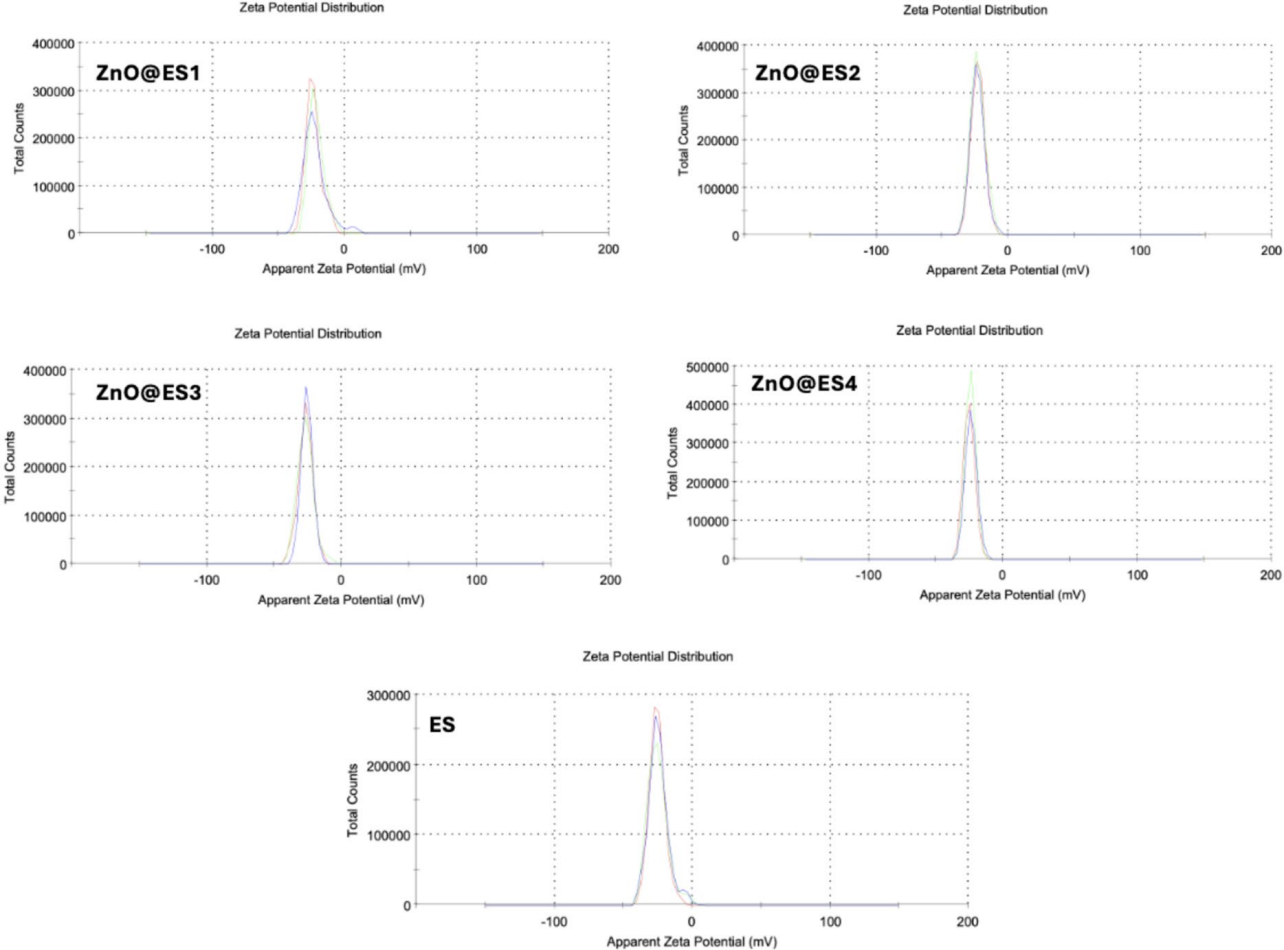
Zeta potential distributions of eggshell powder (ES) and ZnO@ES nanocomposites

Zeta potential is usually used to analyze the mobility, speed and direction of particles suspended in aqueous ionic media with a potential difference between two electrodes immersed in the suspension using electrophoretic kinetic technique (Lunardi et al. 2021; Serrano-Lotina et al. 2023). Zeta potential was calculated using electrophoretic mobility to evaluate the stability of colloidal solutions of ES and ZnO@ES nanocomposites. The distributions of zeta potential for each sample are shown in Fig. 5. While the zeta potential of ES was −24.5, the zeta potentials of ZnO@ES1, ZnO@ES2, ZnO@ES3 and ZnO@ES4 nanocomposites were found to be −22.6, −22.9, −25.6 and −23.7, respectively. All ZnO@ES nanocomposites were observed to have moderate colloidal stability. ZnO loading slightly reduced the natural negative surface charge of the eggshell, indicating that ZnO successfully adhered to the surface and altered the surface chemistry (Wen et al. 2019; Qiu et al. 2020).

### Potential biomaterial properties of synthesized nanocomposites

The compatibility of synthesized nanocomposites with human blood tissue serves as a critical criterion, indicating their potential application as biomaterials in biomedical and pharmaceutical fields.

As shown in Fig. S5 and Table 3, the red blood cell lysis of the synthesized nanocomposites is less than 4%. According to these measurements; ES, ZnO@ES1 and ZnO@ES2 samples can be considered non-hemolytic substances, while ZnO@ES3 and ZnO@ES4 samples can be considered slightly hemolytic substances (Dobrovolskaia et al. 2008a). The ZnO@ES1 nanocomposite demonstrates even lower hemolytic properties compared to eggshell powder, a natural material, highlighting the biocompatibility of materials produced through green synthesis. Hemolysis results indicate that all samples remained within the 5% hemolysis threshold established for biologically safe materials, confirming their hemocompatibility and potential for application as biomaterials in the healthcare sector (Xu et al. 2016).

**Table 3.** Hemolysis values of eggshell powder (ES) and synthesized nanocomposites

Sample	Hemolysis (%)
ES	1.66 ± 0.19
ZnO@ES1	0.88 ± 0.22
ZnO@ES2	2.66 ±0.19
ZnO@ES3	4.00 ± 0.50
ZnO@ES4	3.44 ± 0.44

Results are shown as mean ± standard error of the mean

It is known that zinc oxide nanoparticles alone have antimicrobial effects (Abdelghany et al. 2022; Lyngdoh et al. 2024; Tsakiridou et al. 2024). In many studies, the lethal activity of these nanoparticles has been reported against various microorganisms such as *Vibrio cholerae, Shigella flexneri, Streptococcus mutans, Salmonella typhi, Streptococcus pyogenes, S. aureus, Aspergillus niger, E. coli* and *C. albicans* (Karvani and Chehrazi 2011; Sirelkhatim et al. 2015). Antimicrobial assays were conducted to evaluate the antimicrobial efficacy of nanocomposites synthesized through the green method utilizing zinc oxide nanoparticles, with a focus on identifying which specific nanocomposite exhibits superior antimicrobial properties. As a result of preliminary antimicrobial tests (MIC) performed for four different ZnO@ES nanocomposites synthesized by changing the volumetric ratios of plant extract with zinc nitrate solutions in different molarities, it was determined that nanocomposites ZnO@ES2 and ZnO@ES4 exhibit stronger antimicrobial activity compared to ZnO@ES1 and ZnO@ES3. In line with these findings, it was decided to continue with only ZnO@ES2 and ZnO@ES4 in the further stages of the study.

**Fig. 6:**
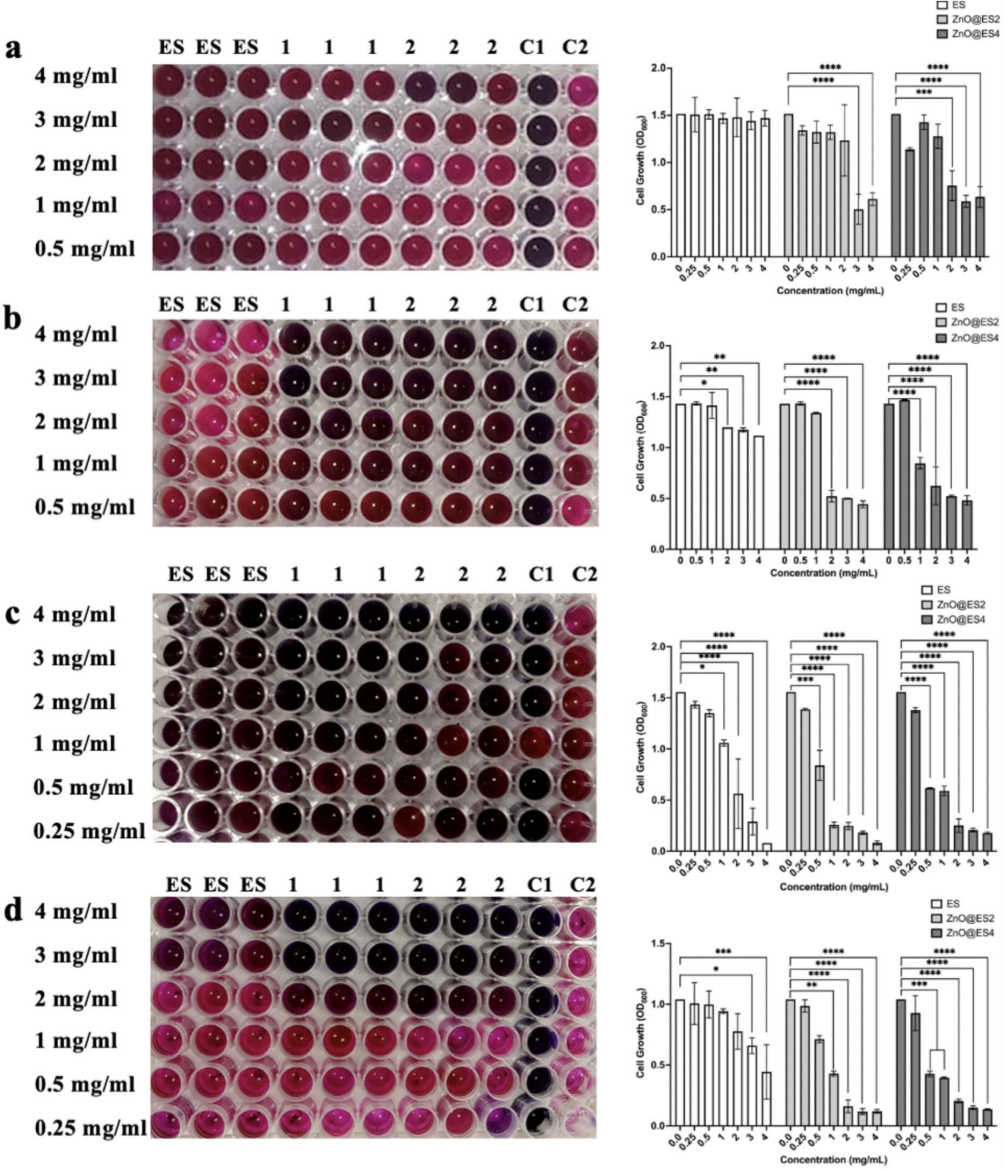
Resazurin dye-based MIC test of eggshell powder (ES) and synthesized nanocomposites against **a** *E. coli* ATCC 35218, **b** *S. aureus* ATCC 25923, **c** *Pseudomonas aeruginosa* ATCC 27853*,* and **d** *C. albicans* ATCC 10239 (ES: eggshell, 1: ZnO@ES4, 2: ZnO@ES2, C1: Dye+Medium, C2: Dye+Culture) Statistical significance level * (p ≤ 0.05), ** (p ≤ 0.01), ***(p ≤ 0.001), **** (p ≤ 0.0001)

The minimum inhibitory concentration (MIC) values of the nanocomposites against *E. coli* ATCC 35218, *S. aureus* ATCC 25923, *P. aeruginosa* ATCC 27853 bacterial strains, and *C. albicans* ATCC 10239 fungus were determined using resazurin dye (Fig. 6). Eggshells exhibited no lethal inhibitory effect against gram-negative *E. coli* bacteria. In contrast, the ZnO@ES2 and ZnO@ES4 nanocomposites demonstrated significant antibacterial effects against *E. coli* at concentrations of 2 and 3 mg/mL, respectively (Fig. 6a). While the eggshell showed a notable inhibitory effect (p ≤ 0.05, p ≤ 0.01) on gram-positive *S. aureus* bacteria within the concentration range of 2–4 mg/mL, it was less effective than the nanocomposites. The ZnO@ES2 and ZnO@ES4 nanocomposites displayed significant MIC effects at concentrations of 2 and 1 mg/mL, respectively (p ≤ 0.0001) (Fig. 6b). The eggshell exhibited a MIC effect at a concentration of 1 mg/mL (p ≤ 0.05), while the ZnO@ES2 (p ≤ 0.001) and ZnO@ES4 (p ≤ 0.0001) nanocomposites were effective at 0.5 mg/mL against gram-negative *P. aeruginosa* bacteria (Fig. 6c). Additionally, although the eggshell (p ≤ 0.05) was effective against *C. albicans* fungus at concentrations up to 3 mg/mL, the ZnO@ES2 (p ≤ 0.01) and ZnO@ES4 (p ≤ 0.001) nanocomposites showed significant MIC effects at concentrations of 1 and 0.5 mg/mL, respectively (Fig. 6d). The results of the MIC tests indicate that the ZnO@ES4 nanocomposite exhibits superior efficacy compared to the ZnO@ES2 nanocomposite. Additionally, it has been noted that high concentrations of eggshell powder may pose toxicity risks to certain bacterial strains. Research has demonstrated the effectiveness of eggshell powder against both gram-positive and gram-negative bacteria, including *S. aureus* and *E. coli* (Wellman-Labadie et al. 2008; Ohshima et al. 2015). This antimicrobial effect is attributed to the detrimental impact of calcium carbonate (CaCO₃) and various organic constituents present in eggshells on microbes (Ohshima et al. 2015). Moreover, eggshell powder has been shown to inhibit bacterial growth by elevating the pH level of the medium (Shang et al. 2022). The MIC findings further reveal that the incorporation of zinc oxide nanoparticles into the eggshell matrix enhances its antimicrobial properties.

The experimental results obtained through the agar well diffusion method indicate that eggshell alone does not demonstrate antimicrobial activity against pathogenic microorganisms, as evidenced by the absence of inhibition zones (Fig. S6). In comparison to the MIC test results, eggshells demonstrated no antibacterial effect in the agar well diffusion test. This lack of efficacy is likely attributed to the greater diffusion of CaCO₃ from the eggshell and the organic compounds within the eggshell membrane structure in a liquid medium, as opposed to a solid agar medium. The ZnO@ES2 nanocomposite exhibited no antimicrobial effect against *S. aureus* and *C. albicans*; however, it did produce inhibition zones against *P. aeruginosa* and *E. coli*. Conversely, the ZnO@ES4 nanocomposite showed no antimicrobial effect against *C. albicans* but effectively inhibited the growth of *P. aeruginosa*, *E. coli*, and *S. aureus*.

Zone diameters were evaluated for various microorganisms using the agar well diffusion method (Table 4). When comparing the inhibition zone diameters of the ZnO@ES2 nanocomposite against various microorganisms, it was observed that the diameters of 14, 10, and 9 mm correspond to the pathogens *E. coli*, *P. aeruginosa*, and *C. albicans*, respectively. Similarly, the ZnO@ES4 nanocomposite exhibited inhibition zone diameters of 14, 9, and 8 mm against *E. coli*, *P. aeruginosa*, and *S. aureus*, respectively. Notably, both ZnO@ES2 and ZnO@ES4 nanocomposites demonstrated equivalent antibacterial effects against *E. coli* and comparable effects against *P. aeruginosa*. However, while the ZnO@ES4 nanocomposite created an inhibition zone against *S. aureus*, the ZnO@ES2 nanocomposite did not. Conversely, the opposite effect was obtained for *C. albicans*.

**Table 4.** Zone diameters (mm) measured against microorganisms in the agar well diffusion method

	*E. coli* ATCC 35218	*P. aeruginosa* ATCC 27853	*S. aureus* ATCC 25923	*C. albicans* ATCC 10239
Chlorhexidine	17	13	16	13
ES	–	–	–	–
ZnO@ES2	14	10	–	9
ZnO@ES4	14	9	8	–

**Fig. 7:**
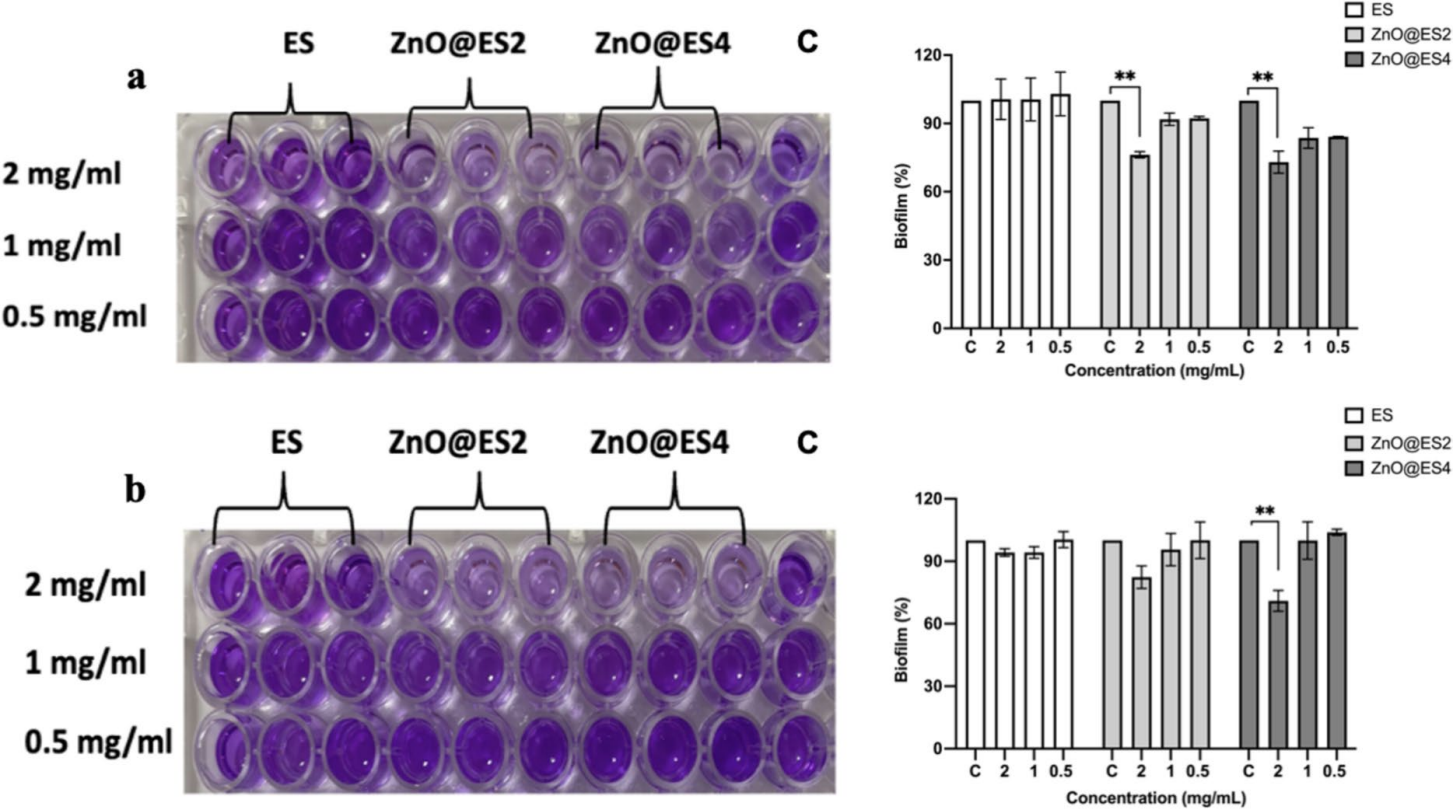
Biofilm formation test for eggshell powder (ES) and synthesized nanocomposites against **a** *E. coli* ATTC 35218 and **b** *P. aeruginosa* ATTC 27853 bacteria. Statistical significance level ** (p ≤ 0.01)

The tested strains of *P. aeruginosa* and *E. coli* are significant bacterial pathogens responsible for various infectious diseases, and they can develop resistance to antibiotics and evade immune responses by forming biofilms on surfaces (Hosnedlova et al. 2022). The biofilm-forming ability of these bacteria complicates treatment efforts. Numerous studies have explored various nanomaterials for biofilm inhibition, highlighting their antimicrobial properties and surface modifications that prevent biofilm formation (Yañez-Macías et al. 2019; Yılmaz et al. 2023). The biofilm formation capacities of *E. coli* ATCC 35218 and *P. aeruginosa* ATCC 27853 were assessed using eggshell powder, ZnO@ES2, and ZnO@ES4 at varying concentrations (2, 1, and 0.5 mg/mL). The evaluation of biofilm inhibitory effects after incubation revealed that eggshell powder did not significantly inhibit biofilm formation. However, the ZnO@ES2 and ZnO@ES4 nanocomposites exhibited the highest biofilm inhibition against *E. coli* at a concentration of 2 mg/mL, with reductions in biofilm formation of approximately 25 and 27%, respectively (p ≤ 0.01) (Fig. 7a). Furthermore, ZnO@ES4 nanocomposite significantly inhibited biofilm formation in *P. aeruginosa* at the same concentration, achieving a 30% reduction (p ≤ 0.01) (Fig. 7b). Although the ZnO@ES2 nanocomposite did not demonstrate a significant inhibitory effect againist *P. aeruginosa* biofilm, it still contributed to a reduction in biofilm formation at 2 mg/mL. Consequently, ZnO@ES4 was determined to be more effective in inhibiting biofilm formation against both bacteria compared to ZnO@ES2.

The antimicrobial activities of the nanocomposites are primarily attributed to the effects of ZnO nanoparticles, as demonstrated by MIC, agar diffusion, and biofilm inhibition tests. Several mechanisms contribute to the antimicrobial efficacy of ZnO nanoparticles synthesized via green synthesis within the eggshell structure (Qi et al. 2017; Suvaitha et al. 2022). These mechanisms result from a combination of multiple biophysical and biochemical interactions. ZnO nanoparticles generate reactive oxygen species (ROS) on their surface, particularly hydroxyl radicals (•OH), superoxide anions (O₂•⁻), and hydrogen peroxide (H₂O₂), which damage microbial cell membranes, disrupt membrane permeability, and cause intracellular leakage. This oxidative stress compromises the structural integrity of key cellular components such as DNA, proteins, and lipids, ultimately leading to cell death. Additionally, the release of Zn^2^⁺ ions further enhance antimicrobial activity. These ions disrupt the ionic balance across microbial membranes, impair metabolic functions, and inhibit enzymatic systems. ZnO nanoparticles can also interact electrostatically with bacterial and fungal cell membranes, disrupting membrane integrity and causing leakage of cellular contents (Zhang et al. 2019; Selvam et al. 2021). The eggshell serves a dual role. It provides physical support that promotes more uniform dispersion of ZnO nanoparticles and helps regulate surface pH, thereby optimizing the activity of ZnO (Wellman-Labadie et al. 2008; Ohshima et al. 2015). In conclusion, the antimicrobial activity of ZnO@ES nanocomposites arises from the synergistic effects of multiple mechanisms, including ROS generation, Zn^2^⁺ ion release, membrane interactions, and the supportive role of the ES matrix.

**Fig. 8:**
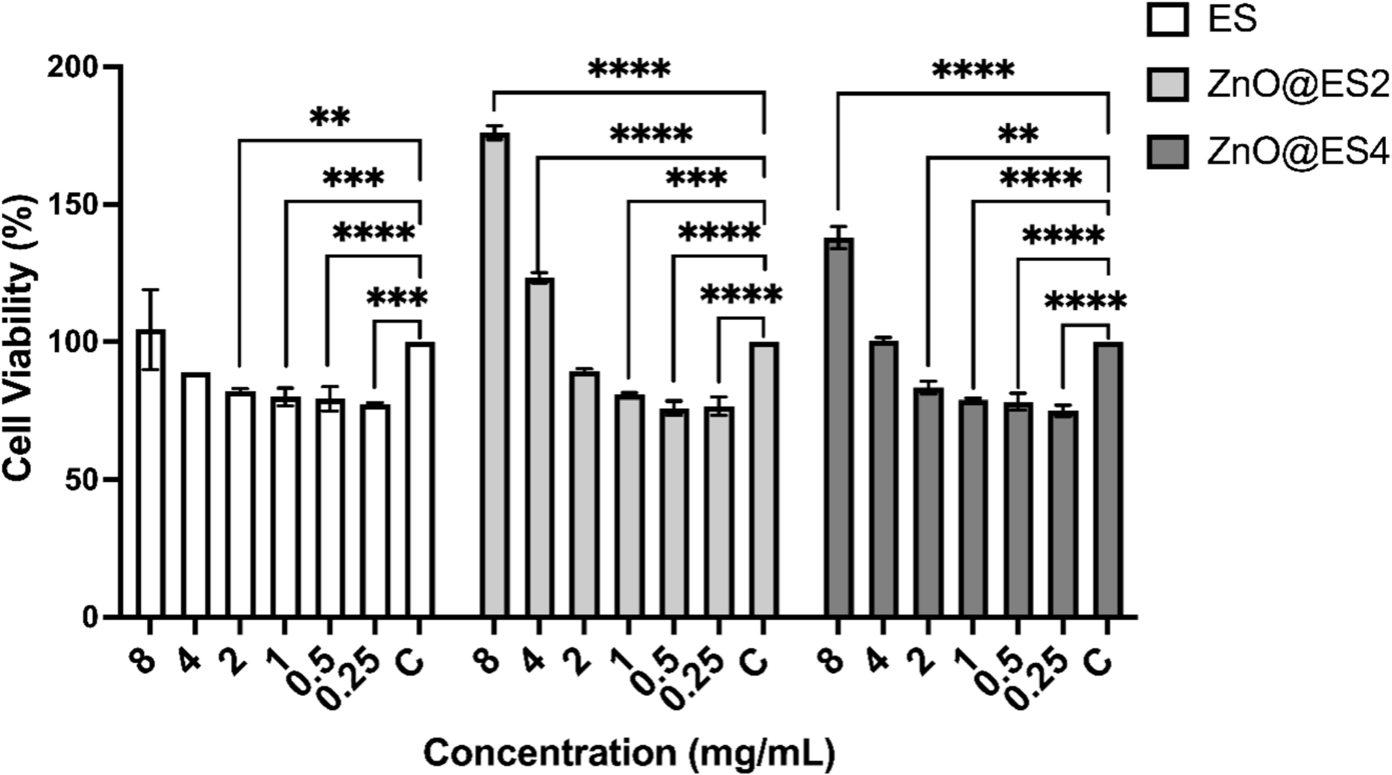
MTT cytotoxicity test for eggshell powder (ES) and synthesized nanocomposites against L929 fibroblast cell line. Statistical significance level ** (p ≤ 0.01), ***(p ≤ 0.001), **** (p ≤ 0.0001)

The results of the MTT cytotoxicity assay for ZnO@ES2 and ZnO@ES4 nanocomposite samples, evaluated at various concentrations on the L929 fibroblast cell line, are illustrated in Fig. 8. It was noted that cell viability exhibited an upward trend with increasing sample concentrations in the MTT assay. The control group, representing cell viability at 0 mg/mL for each sample, served as the baseline reference. ZnO@ES2 demonstrated a marked enhancement in cell viability with increasing concentrations relative to the control group, achieving the highest viability at 8 mg/mL, which corresponded to a significant increase (76%) in cell proliferation (p ≤ 0.0001). ZnO@ES4 also indicated a significant rise in cell viability at high concentrations compared to the control group, with a 38% increase in cell proliferation at 8 mg/mL (p ≤ 0.0001). Conversely, eggshells alone exhibited a more suppressive effect at lower concentrations (0.25–2 mg/mL) when compared to the control group, resulting in decreased cell viability. Although a non-significant increase in cell viability was observed at higher concentrations (4 and 8 mg/mL) for eggshells, this increase was not as pronounced as that seen with ZnO@ES2 and ZnO@ES4. In summary, the ZnO@ES2 and ZnO@ES4 nanocomposites demonstrated superior biocompatibility and a favorable profile regarding cell viability in comparison to the control group, while eggshells exhibited a more toxic effect, particularly at lower concentrations. The cytotoxicity assessment conducted on L929 fibroblast cells indicated that the ZnO@ES2 nanocomposite outperformed ZnO@ES4 in promoting cell proliferation.

The results of the MTT cytotoxicity assay align with the findings in the literature, highlighting the biocompatibility and cell viability-enhancing effects of zinc oxide-based nanocomposites. It is well-documented that zinc oxide nanoparticles support cellular metabolic processes by releasing zinc ions, which play a pivotal role in enzyme activation, cell signaling, and proliferation (Rasmussen et al. 2010; Ahamed et al. 2008). In this study, the ZnO@ES2 and ZnO@ES4 nanocomposites demonstrated a consistent increase in cell viability with rising concentrations, reaching their highest values at 8 mg/mL. These findings are consistent with previous studies, which suggest that these nanoparticles synthesized via green methods benefit from natural stabilizing agents, thereby reducing toxicity and enhancing biocompatibility (Dobrovolskaia et al. 2008b). Moreover, green synthesis approaches are reported to regulate reactive oxygen species (ROS) production, minimizing cellular stress and cytotoxicity while promoting cell survival (Tang and Lv 2014). In contrast, the eggshells alone exhibited a suppressive effect on cell viability at lower concentrations, likely due to the absence of bioactive zinc ions and limited inherent biological activity. This observation underscores the variability in biocompatibility and toxicity profiles among biomaterials, driven by their compositional and synthesis-specific characteristics, as highlighted in the literature (Dobrovolskaia et al. 2008b; Tang and Lv 2014).

## Conclusion

This research details the green synthesis of zinc oxide nanoparticles using eggshells and *Althaea officinalis* flower extract, a sustainable and cost-effective method. Evaluating the HPLC analysis demonstrated that the pink flower extract has the highest quercetin content, making it the best choice for nanocomposite synthesis. During synthesis, the color changed from pink to greenish-light brown indicating zinc oxide nanoparticle formation. Moreover, the characteristic absorbance peak at 384 nm confirmed the presence of zinc oxide nanoparticles by UV–Vis absorption spectroscopy.

The resulting nanocomposites (ZnO@ES) were further characterized by ATR-FTIR, XRD, SEM-EDX, and TEM analyses. FTIR spectral analysis confirmed the presence of calcite and membrane proteins in the eggshell powder. Zinc oxide-related IR bands around 600 and 400 cm^−1^, and specifically at 480 and 499 cm^−1^ indicate formation and interaction between the zinc oxide nanostructure and the eggshell powder. Therefore, the successful immobilization of zinc oxide nanoparticles within the eggshell matrix was achieved without disrupting the calcite and membrane structures. XRD patterns confirmed the presence of both ZnO and CaCO_3_ from eggshells, indicating the formation of the nanocomposite structure. SEM and TEM imaging showed that the porous eggshell structures were coated with zinc oxide and these nanoparticles formed various morphologies, including nanorods, nanoflakes, and nanoparticles, with sizes ranging from 24 to 50 nm, suitable for nanomedicine applications. EDX analysis further confirmed the presence of Zn, Ca, C, O, S, and Mg in the nanocomposites, supporting the successful formation of zinc oxide. Zeta potential results indicated that the synthesized nanocomposites exhibit moderate stability and confirmed the successful loading of zinc oxide nanoparticles onto the eggshell.

The study investigated the biocompatibility and antimicrobial properties of the nanocomposites through hemolysis, MIC, agar well diffusion, and biofilm inhibition tests. Finally, cytotoxicity assays using L929 fibroblast cells assessed the nanocomposites' biocompatibility, revealing their potential as a biomaterial for biomedical applications. All nanocomposites showed hemolysis rates under 4%, indicating good hemocompatibility. ZnO@ES2 and ZnO@ES4 nanocomposites showed significant antimicrobial activity against *E. coli, S. aureus, P. aeruginosa*, and *C. albicans* in the MIC test. The eggshell alone showed limited antimicrobial activity. In the case of the agar well diffusion test, ZnO@ES2 showed effectiveness against *E. coli* and *P. aeruginosa*, while ZnO@ES4 inhibited *E. coli, P. aeruginosa*, and *S. aureus*. No inhibition zone was observed with eggshell alone. Both ZnO@ES2 and ZnO@ES4 significantly reduced biofilm formation in *E. coli* and *P. aeruginosa*, with ZnO@ES4 showing higher inhibition capabilities. ZnO@ES2 and ZnO@ES4 showed no cytotoxicity to L929 fibroblast cells and increased cell proliferation at higher concentrations. The eggshell alone had a more suppressive effect at lower concentrations.

ZnO@ES2 and ZnO@ES4 nanocomposites are advantageous for biomaterial applications due to their high biocompatibility and cell viability support properties. They offer safe and effective potential use, especially in biomedical fields such as wound healing and tissue engineering. They have potential biomaterial features in healthcare by adding antimicrobial properties to creams, ointments, and even bandages as a filling material in hydroxyapatite-rich tooth and bone structures. The study underscores the importance of sustainable and environmentally friendly approaches in biomaterial science holding potential medical applications.

## Supplementary Information

Below is the link to the electronic supplementary material.

Supplementary file1 (DOCX 3379 KB)

## Data Availability

No datasets were generated or analysed during the current study.
