# Supplementary material for: Biofilm-inhibiting ZnO@Eggshell nanocomposites: green synthesis, characterization, and biomedical potential
Source: Biometals. 2025 Jul 2;38(5):1447–68. doi: 10.1007/s10534-025-00711-8 (PMC12507985; doi:10.1007/s10534-025-00711-8)
Supplement: Supplementary file 1 — Supplementary file1 (DOCX 3379 KB) [file 10534_2025_711_MOESM1_ESM.docx]

# **Supplementary Figures**


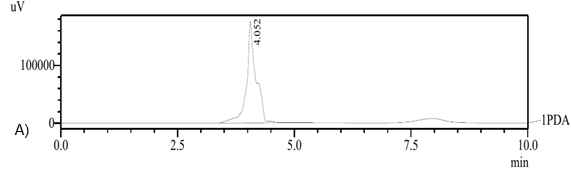


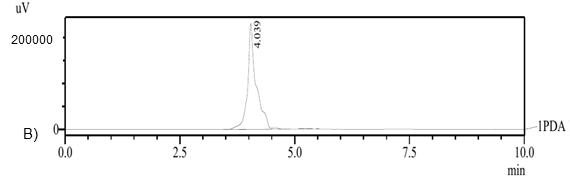


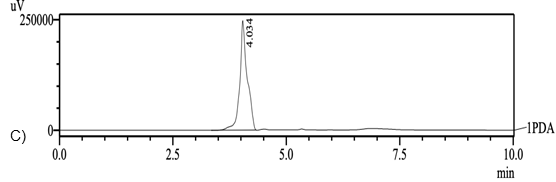


**Figure S1.** HPLC chromatograms of quercetin in **a)** reddish pink flower extract **b)** white flower extract, and **c)** pink flower extract obtained from *Althaea officinalis*


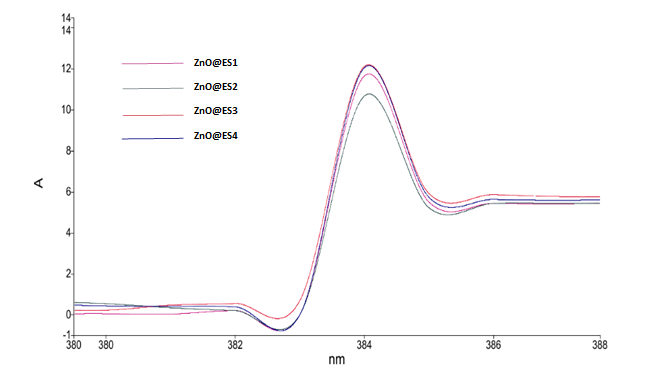


**Figure S2** UV-Vis spectra showing maximum absorbance at λ384 nm for zinc oxide nanoparticles synthesized with pink flower extract of *Althaea officinalis*

*
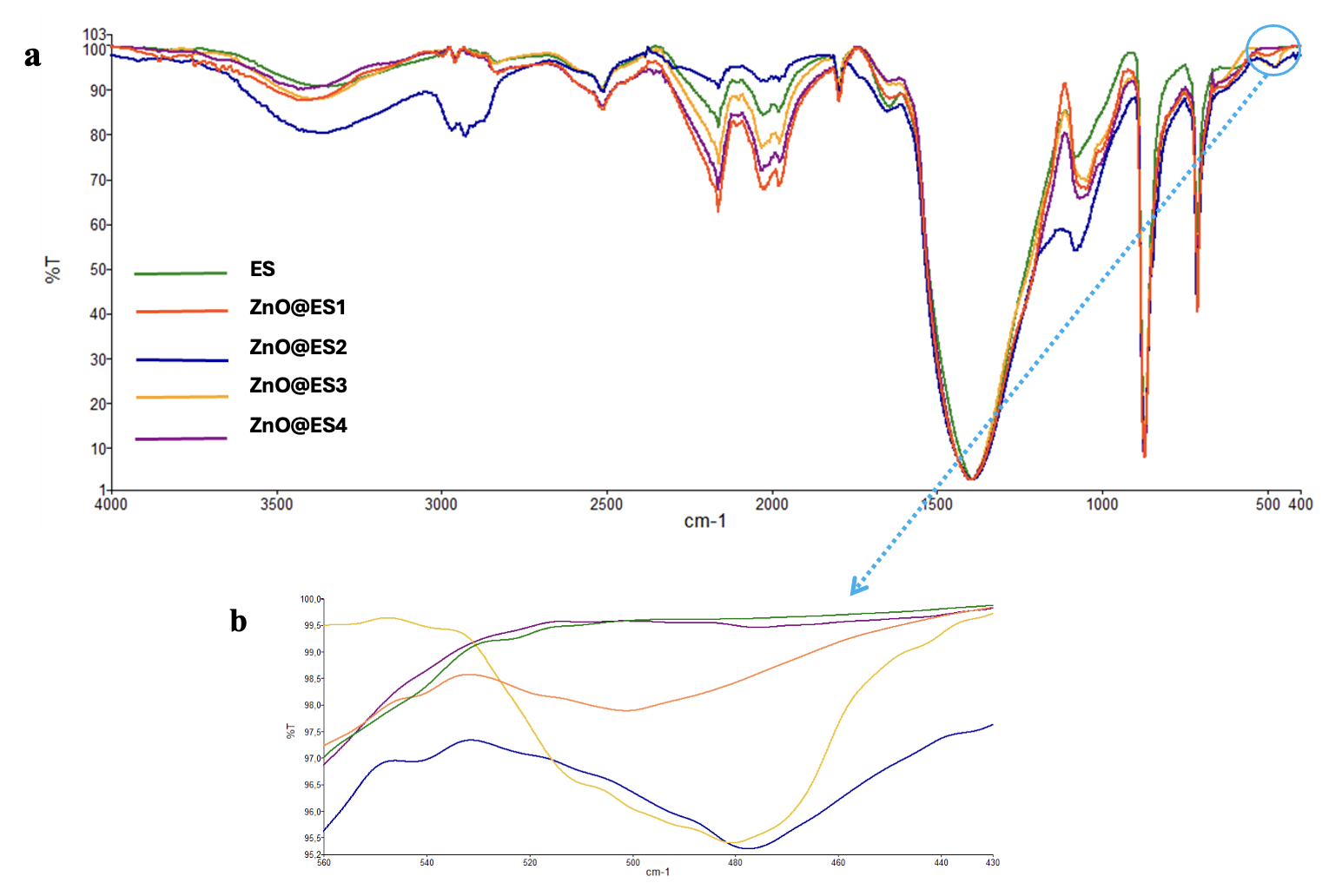
*

**Figure S3.** **a)** FTIR spectra of empty eggshell powder (ES), ZnO@ES1, ZnO@ES2, ZnO@ES3 and ZnO@ES4 nanocomposites in the 4000-400 cm^-1^ spectral region. **b)** The enlarged panel in the 550-450 cm^-1^ spectral region demonstrating the absorption band gap associated with the metal-oxygen stretching mode in zinc oxide


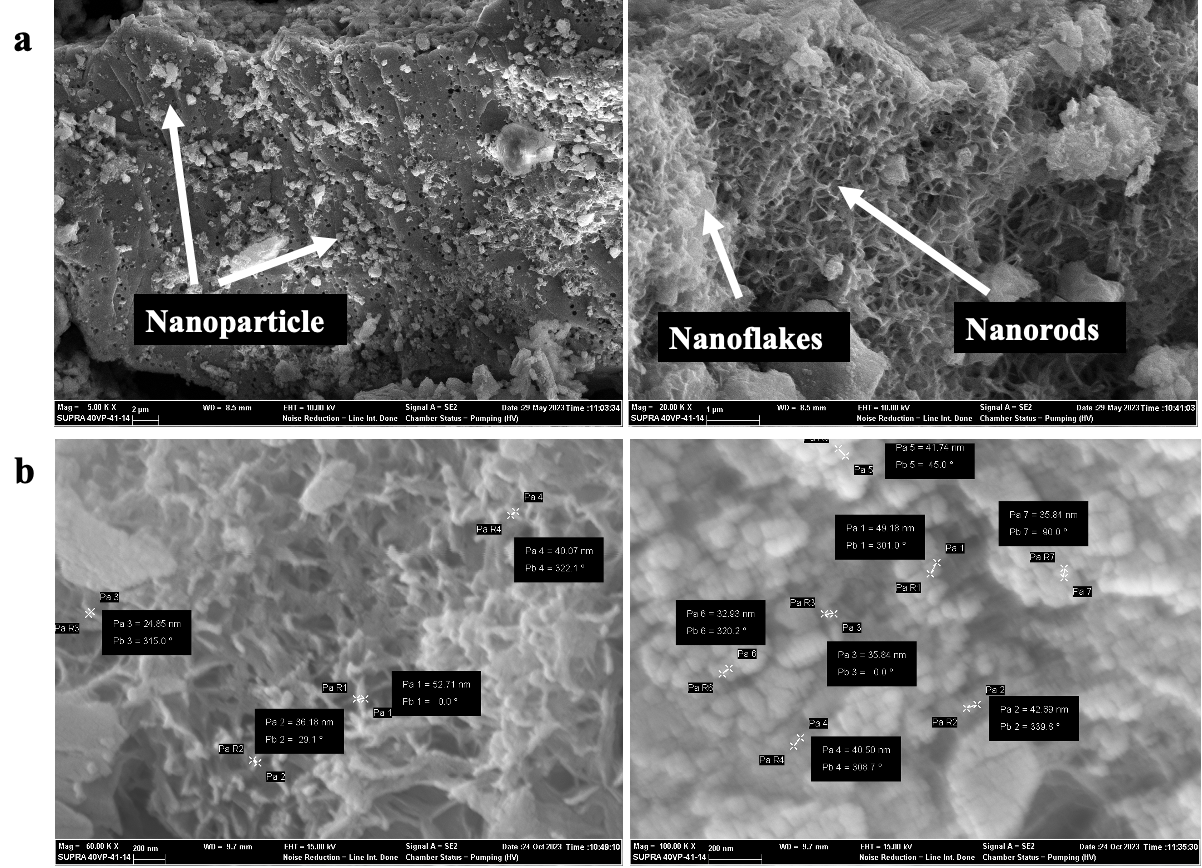


**Figure S4. a)** Different ZnO nanostructures **b)** dimensions of ZnO nanorods and ZnO nanoparticles (nm)


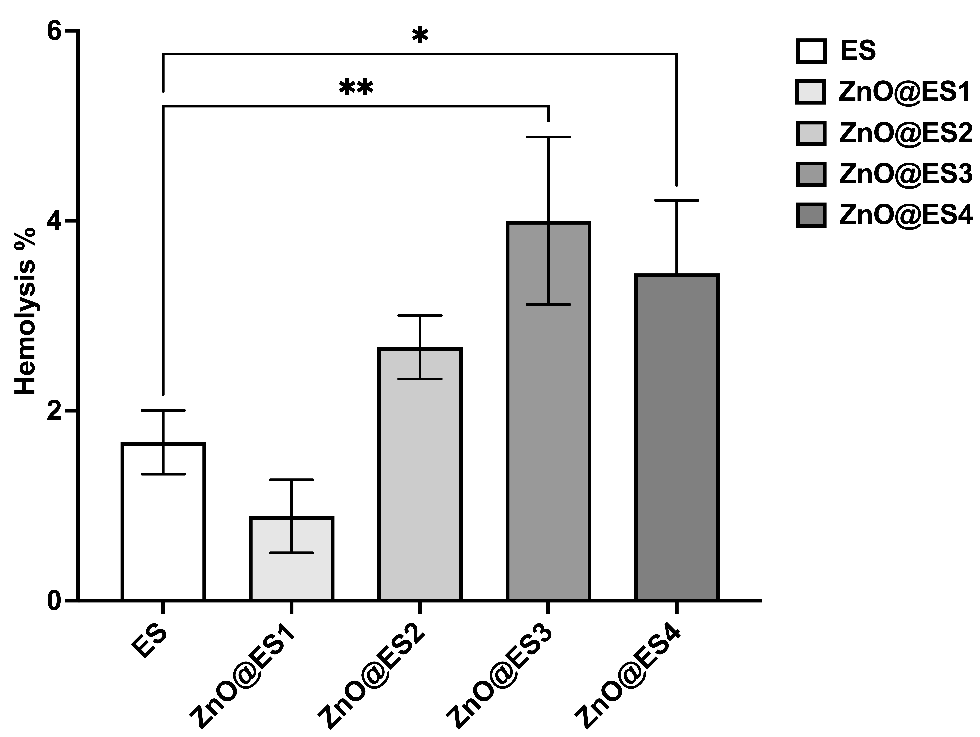


**Figure S5.** Hemolysis values ​​of eggshell powder (ES) and synthesized nanocomposites. Results are shown as mean value ± standard error of the mean. Statistical significance level * and ** (p≤0.05 and p≤0.01)


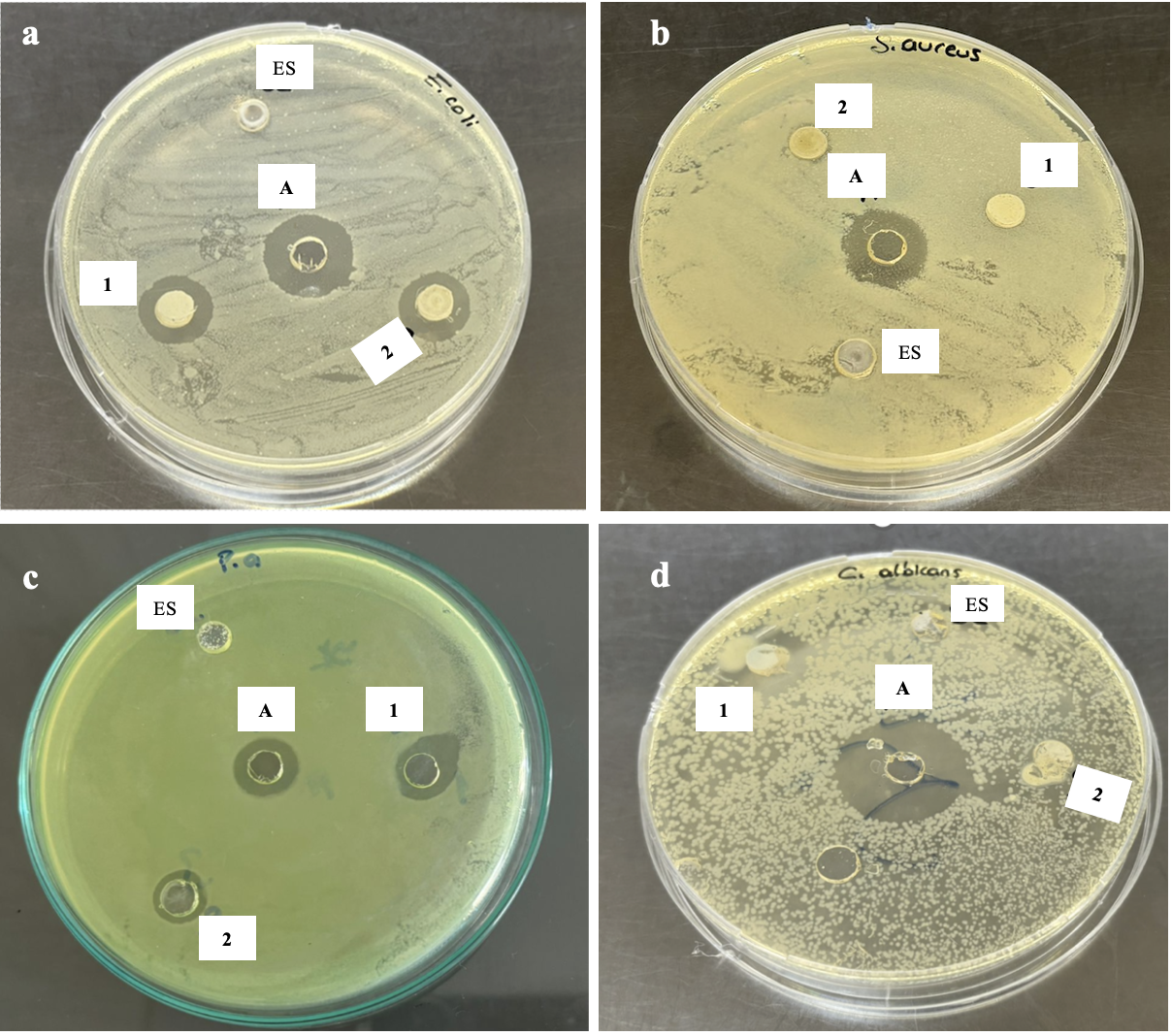


**Figure S6**. Agar well diffusion test for eggshell powder (ES) and synthesized nanocomposites against **a)** *Escherichia coli* ATCC 35218, **b)** *Staphylococcus aureus* ATCC 25923, **c)** *Pseudomonas aeruginosa* ATCC 27853*,* and **d)** *Candida albicans* ATCC 10239. Chlorhexidine (CHX) was used as a positive control (A: CHX, 1: ZnO@ES2, 2: ZnO@ES4, ES: eggshell)
